# Overexpression of PAX8-AS1 Inhibits Malignant Phenotypes of Papillary Thyroid Carcinoma Cells via miR-96-5p/PKN2 Axis

**DOI:** 10.1155/2021/5499963

**Published:** 2021-10-26

**Authors:** Ping Zhou, Tongdao Xu, Hao Hu, Fei Hua

**Affiliations:** ^1^Department of Endocrine, The Second People's Hospital of Lianyungang, Lianyungang 222000, Jiangsu, China; ^2^Department of Endocrine, The Third Affiliated Hospital of Soochow University, Changzhou 213003, Jiangsu, China

## Abstract

**Background:**

Thyroid carcinoma (THCA) is the most frequent endocrine malignancy. Papillary thyroid carcinoma (PTC) is the major subtype of THCA, accounting for over 80% of all THCA cases. LncRNA PAX8-AS1, a tumor suppressor associated with various human cancers, has been reported to be relevant to the regulation of all sorts of cellular processes. The purpose of this study was to verify the role of PAX8-AS1 in PTC.

**Methods:**

Three human PTC cell lines (K1, TPC-1, and IHH4) and one normal human thyroid cell line, Nthy-ori3-1, were used in our study. The expression of genes was detected by qRT-PCR. The bioinformatic analysis and luciferase reporter assay were used to confirm the binding relationship of PAX8-AS1 to miR-96-5p, and the targeting relationship of miR-96-5p to PKN2 was also predicted. Cell proliferation and apoptosis capacities were assessed by MTT and flow cytometry, respectively. EdU assay was used to detect cell proliferation. Western blot assay was employed to examine protein expression.

**Results:**

The expression of PAX8-AS1 was decreased in PTC tissues and cells. PAX8-AS1 overexpression inhibited the proliferation of PTC cells and promoted cell apoptosis. In addition, PAX8-AS1 bonds with miR-96-5p, whose downregulation elevated the expression of PKN2 in PTC cells. Importantly, according to the rescue experiments, PKN2 silencing partially reversed the inhibitory effects of PAX8-AS1 expression on PTC cell proliferation and apoptosis.

**Conclusions:**

We found that the PAX8-AS1/miR-96-5p/PKN2 axis was closely related to the progression of PTC, which could be a potential target for treating PTC patients.

## 1. Introduction

Over the past three decades, an increasing number of people worldwide have been diagnosed with thyroid carcinoma (THCA) [1]. About 567,000 new cases were reported worldwide both in men and women in 2018 [2]. Surgery is the most common treatment for THCA. In addition to surgery, common medical treatments include chemotherapy and radioactive iodine [3–5]. However, the above treatments cannot deal with the prognosis of recurrent THCA [6, 7]. Papillary thyroid carcinoma (PTC) is the major subtype of THCA, accounting for over 80% of all THCAs [8]. PTC patients usually suffer from metastasis to lymph nodes and lungs, resulting in poor clinical outcomes [9]. Genetic and epigenetic alterations have been demonstrated to play pivotal roles in the development of PTC [10]. Therefore, clarifying the molecular mechanisms underlying the development of PTC is important to improve the therapeutic strategies for PTC.

Long noncoding RNAs (lncRNAs) are an important component of the newly discovered noncoding RNAs (lncRNAs) [11]. LncRNAs can act as a tumor inhibitor or an oncogene [12]. Studies have indicated that lncRNAs make a difference in the process of tumorigenicity, proliferation, migration, apoptosis, and differentiation. Some lncRNAs have been determined as a major contributor to the development of PTC, and these lncRNAs are urgently needed as new biomarkers for early diagnosis and even treatment [13–15]. As a potential regulator of PAX8, PAX8 antisense RNA 1 (PAX8-AS1) contains certain single nucleotide polymorphisms (SNPs) which represents expression quantitative trait loci (eQTL) for PAX8 [16]. Only a few previous studies have assessed the effect of PAX8-AS1 variants on cancer risk. In detail, it has been reported that rs4848320 and rs1110839 PAX8-AS1 variants significantly reduce the risk of cervical cancer [17]. Zhang et al. identified that PAX8-AS1 regulates the hub genes in triple-negative breast cancer cell [18]. Particularly, the expression level of PAX8-AS1 was shown to be significantly associated with overall or recurrence-free survival time in patients with PTC [19]. Nevertheless, no previous study has investigated the regulatory mechanism of PAX8-AS1 in PTC.

Competitive endogenous RNA (ceRNA) network, which represents a new mechanism of interaction between RNAs, plays a crucial role in multiple biological processes and development of tumors [20]. LncRNAs can serve as ceRNAs, which competitively bind micro-RNAs (miRNAs) to inhibit its regulation of downstream mRNAs [21]. As we all know, miRNAs, as a small class of noncoding single-stranded RNAs, play a pivotal role in the malignant progression of different tumors [22]. According to the Encyclopedia of RNA Interactomes (ENCORI) website (http://starbase.sysu.edu.cn/), miR-96-5p was found to have binding sites with PAX8-AS1. miR-96-5p is a member of the miR-183-96-182 cluster, which plays an indispensable role in tumor regulation [23]. Previously, miR-96-5p was reported to promote the development of many human cancers including PTC. For example, miR-96-5p is upregulated in human oral cancer tissues compared to normal adjacent tissues and has been identified in studies as a cancer-causing gene and biomarker for oral cavity tumor [24]. In addition, miR-96-5p overexpression enhances colorectal cancer cell migration and invasion, playing a carcinogenic role in the development of colorectal cancer [25]. Importantly, miR-96-5p is upregulated in PTC tissues and cells, and miR-96-5p overexpression promotes proliferation, invasion, and migration of PTC cells via targeting CCDC67 [26]. Since the ceRNA role of PAX8-AS1 has been reported in breast cancer [27], we suspected that PAX8-AS1 might act as a ceRNA against miR-96-5p in PTC.

In our research, we verified the function of PAX8-AS1 in PTC and demonstrated that overexpressed PAX8-AS1 could inhibit the malignancy of PTC cells through the miR‐96-5p/PKN2 axis. Our study suggests that PAX8-AS1 may serve as a potential novel prognostic biomarker and therapeutic target for PTC treatment.

## 2. Materials and Methods

### 2.1. Cell Culture

Human PTC cell lines (K1, TPC-1, and IHH4) and normal human thyroid cell line, Nthy-ori3-1, were purchased from American Tissue Culture Collection (ATCC, Manassas, VA, USA). All cells were cultured in the Roswell Park Memorial Institute (RPMI) 1640 medium with 10% fetal bovine serum (FBS, Gibco Company) in a humidified atmosphere with 5% CO_2_ at 37°C. The medium was replaced every 2-3 days. The subculture was performed when the degree of cell fusion was up to 80% to 90%.

### 2.2. Cell Transfection

Expression of lncRNA PAX8-AS1 in PTC cells was overexpressed by pcDNA3.1/PAX8-AS1 plasmid (PAX8-AS1), and empty pcDNA3.1 (vector) served as control. Short hairpin RNA (shRNA) specifically targeting PKN2 (sh-PKN2) was used to downregulate the PKN2 expression in PTC cells. The miR-96-5p inhibitor was used to knockdown miR-96-5p, with NC inhibitor as the negative control. All these plasmids were purchased from GenePharma (Shanghai, China) and transfected into K1 and IHH4 cells with Lipofectamine 2000 (Invitrogen, Carlsbad, CA, USA) according to manufacturer's instructions. Transfection efficiency was detected by quantitative real-time polymerase chain reaction (qRT-PCR).

### 2.3. Quantitative Real-Time Polymerase Chain Reaction (qRT-PCR)

Total RNA was extracted from cell culture samples using PrimeScriptTM RT Master Mix and PrimeScriptTM RT Reagent Kit (Takara, Shiga, Japan). Then, the complementary DNA (cDNA) was formed through quantifying and reverse transcribing using the First Strand cDNA Synthesis Kit (Fermentas, Burlington, Canada). Real-time PCR was performed using the SYBR premix Ex Taq kit (Takara) on Applied Rotor-Gene 6000 Real Time PCR System (Corbett Research, Mortlake, Australia). After the reaction, the threshold cycle (Ct) was measured. The experiment was conducted three times, and the expressions of RNA were evaluated with the 2^−ΔΔCt^ method. For lncRNA and mRNA expression, paired primers (Fwd and Rev) were used with GAPDH mRNA for normalization. For miRNA expression, paired primers (fwd and rev) were used with the level of U6 snRNA as an endogenous reference gene. The primer sequences are listed in Table 1.

### 2.4. 3-(4,5)-Dimethylthiahiazo(-z-y1)-3,5-diphenytetrazoliumromide (MTT) Assay

MTT kit (Dojindo, Kumamoto, Japan) was used to detect cell proliferation. MTT assay was used to dilute the cell suspension and inoculate cells with 5 × 10^4^ cells per well into 96-well plates. Six wells were prepared for each group to repeat. As soon as the degree of cell fusion reached 80%, the cells were treated according to the above groups. MTT solution (20 *μ*l, Sigma Aldrich, St. Louis, MO, USA) was added after cultured for 24 h, 48 h, and 72 h. After incubation at 37°C for 4 h, MTT solution was discarded. Then, dimethyl sulfoxide (DMSO, 150 *μ*l, Sigma Aldrich, St. Louis, MO, USA) was added to each well. The OD value of each well was measured at 490 nm with a microplate analyzer after shaking the plate for 10 min. The experiment was repeated three times, and the average OD value was calculated.

### 2.5. 5-Ethynyl-2′-eoxyuridine (EdU) Assay

EdU labeling and detection kit (Ribobio, Guangzhou, China) was used to detect cell proliferation. Cells were cultured in 96-well plates at the density of 5 × 10^3^ cells/well. After transfecting for 48 h, the 96-well plates as added with 50 *μ*M EdU labeled media and incubated at 37°C for 2 h with 5% CO_2_. Cells were treated with 4% paraformaldehyde and 0.5% Triton X-100, and cell nuclei were labeled with anti-EDU working fluid DAPI. After the fluorescent microscopy analysis, the percentage of EdU-positive cells was calculated. Each treatment group was randomly assessed for five fields of view.

### 2.6. Flow Cytometry Analysis

The cells were treated with trypsin by flow cytometry, and then the cells were collected at the end of treatment and centrifuged for 5 min at 1000 rpm to extract the supernatant. The cells were resuspended and rinsed with PBS, and in order to prepare single-cell suspension, the cell density was adjusted to 1 × 10^6^ cells/mL. Each group was added with 500 *μ*L cold ethanol of about 70% fraction, fixed for 2 h at 4°C, and left to rest overnight. The fixed solution was discarded, 1 ml PBS was added to rinse the fixed solution, the supernatant was extracted, and Annexin V-FITC was added after the centrifugation at 2000 rpm for 3 min, and propidium iodide (PI) stain liquid was added subsequently. After dark culture at 4°C for 30 min, flow cytometer was used for detection.

### 2.7. Western Blot Analysis

Total protein samples in PTC cells were extracted by radioimmunoprecipitation buffer (Beyotime, Shanghai, China) with a mixture of protease inhibitors. The same amounts of protein samples were placed into 12% polyacrylamide gel wells, and electrophoresis was performed. The protein samples were then transferred onto nitrocellulose blotting membranes and sealed tightly with 5% fat-free milk. Then, the membranes were incubated with the primary antibodies (Abcam, Cambridge, UK) against PKN2 (ab138514), cleaved caspase 3 (ab32042), Bax (ab32503), Bcl-2 (ab32124), and GAPDH (ab8245) at 4°C for 12 h. The protein bands were analyzed using the Odyssey R Infrared Imaging System (LI-COR Biosciences) after incubating with IRDye R secondary antibody.

### 2.8. Luciferase Reporter Assay

The wild type or mutant PAX8-AS1 sequences and 3′ untranslated region (UTR) of PKN2 were subcloned downstream of the luciferase gene in the pmirGLO luciferase reporter vector (Promega, Beijing, China). PTC cells were seeded into 24-well plates. After overnight incubation, PTC cells were cotransfected with 150 ng of NC inhibitor or miR‐96-5p inhibitor and 50 ng of a firefly luciferase reporter comprising wild type or mutant PAXA-AS1 (or 3′ UTR of a PKN2 fragment) using Lipofectamine 3000 (Invitrogen). Forty-eight hours after transfection, the luciferase activity was determined using the Dual-Luciferase Kit (Promega). The relative firefly luciferase activities were normalized to those of Renilla luciferase.

### 2.9. Statistical Analysis

All experiments were repeated in triplicate. The mean ± standard deviation was used to express data of normal distribution. Median values are used to represent data that are not normally distributed. The difference between two groups of normal distribution data was analyzed by Student's *t*-test, and the difference between multiple groups was analyzed by one-way analysis of variance (ANOVA) and Tukey's *post hoc* tests. SPSS 21.0 was used for statistical analysis. *P* values <0.05 were considered statistically significant.

## 3. Result

### 3.1. LncRNA PAX8-AS1 Shows Low Expression in PTC Tissues and Cells

**Figure 1:**
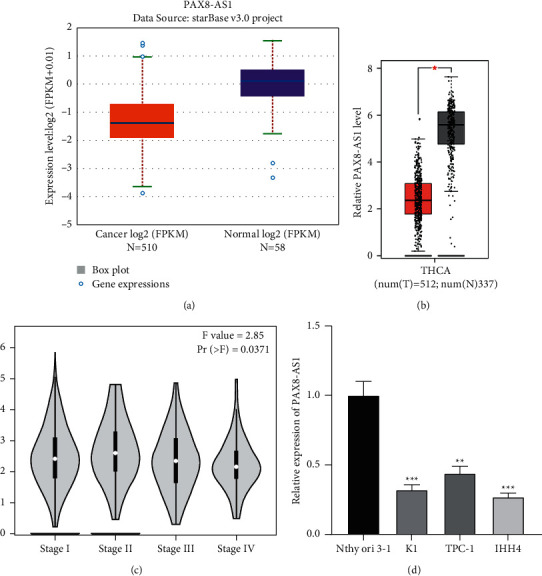
Low expressed lncRNA PAX8-AS1 in PTC tissues and cells. *Note.* (a) ENCORI website (http://starbase.sysu.edu.cn/) showed the expression of PAX8-AS1 in THCA tissues (*n* = 510) and normal tissues (*n* = 58); (b) GEPIA website (http://gepia2.cancer-pku.cn/) showed the expression levels of PAX8-AS1 in THCA tissues (*n* = 512) and normal tissues (*n* = 337); (c) GEPIA website showed the expression level of PAX8-AS1 in different stages of THCA patients; (d) the expression of PAX8-AS1 in PTC cell lines (K1, TPC-1, and IHH4) and human normal thyroid Nthy-ori 3-1 cells. ^*∗*^*P* < 0.05, ^*∗∗*^*P* < 0.01,  and ^*∗∗∗*^*P* < 0.001.

The expression level of PAX8-AS1 in the PTC tissues and cells was determined by qRT-PCR. First, 510 PTC tissues showed lower expression of PAX8-AS1 than 58 normal tissues according to ENCORI website (http://starbase.sysu.edu.cn/) (Figure 1(a)). Similarly, the PAX8-AS1 expression level in 512 PTC tissues were lower than in 337 control normal tissues in the Gene Expression Profiling Interactive Analysis (GEPIA) dataset (http://gepia2.cancer-pku.cn/) (Figure 1(b)). Based on the stage plot (Figure 1(c)), expression of PAX8-AS1 was lower in PTC tissues at stage III and stage IV, relative to those at stage I and stage II. Together, these results suggest that the expression of PAX8-AS1 is clearly associated with the development of PTC. Consistently, low PAX8-AS1 expression was shown in PTC cells (K1, TPC-1, and IHH4), compared to human normal thyroid Nthy-ori 3-1 cells (Figure 1(d)). Importantly, PTC IHH4 cells and K1 cells had the lowest expression of PAX8-AS1 and were most significantly different from Nthy-ori 3-1 cells. Thus, we selected K1 and IHH4 cells for the following experiments. These results indicated that PAX8-AS1 is downregulated in PTC.

### 3.2. LncRNA PAX8-AS1 Impedes PTC Cell Proliferation and Enhances Apoptosis

**Figure 2:**
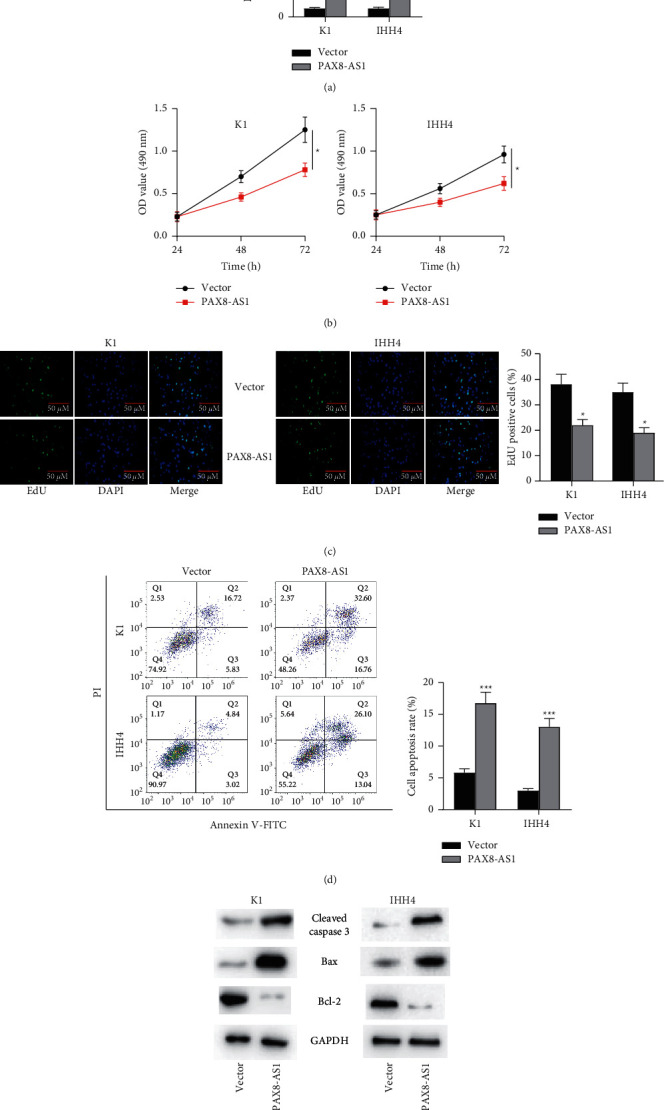
Overexpression of PAX8-AS1 impedes the cell proliferation and enhances apoptosis. *Note.* (a) The transfection efficiency of PAX8-AS1 in PTC K1 and IHH4 cells was detected by qRT-PCR; (b) MTT assay was conducted to separately detect the optical density values at 490 nm at 24, 48, and 72 h in K1 and IHH4 cells after overexpressing PAX8-AS1; (c) cell proliferation was examined in K1 and IHH4 cells after PAX8-AS1 overexpression by EdU assay; (d) flow cytometry was used to analyze the scatter patterns of cell cycle distribution of K1 and IHH4 cells after PAX8-AS1 overexpression; (e) the protein bands of apoptosis-related proteins in K1 and IHH4 cells were detected by western blot. ^*∗*^*P* < 0.05,  ^*∗∗*^*P* < 0.01, and ^*∗∗∗*^*P* < 0.001.

Proliferation and apoptosis are identified as important indexes of various cancers [28]. To test how they are influenced by PAX8-AS1, we overexpressed it in K1 and IHH4 cells. The transfection efficiency was examined by qRT-PCR (Figure 2(a)). According to the MTT assay (Figure 2(b)), compared with the vector group, the proliferation of K1 and IHH4 cells was inhibited in the PAX8-AS1 group, which suggested that PAX8-AS1 overexpression suppressed PTC cell proliferation. Furthermore, EdU assay also demonstrated that K1 and IHH4 cells displayed poor proliferation abilities after overexpressing PAX8-AS1 (Figure 2(c)). Taken together, PAX8-AS1 overexpression suppressed PTC cell proliferation. Flow cytometry was used to detect the apoptosis of K1 and IHH4 cells (Figure 2(d)). In both cells, the PAX8-AS1 group showed a higher cell apoptosis rate than the vector group. The expression of cleaved caspase 3, Bax, and Bcl-2 in K1 and IHH4 cells was examined by western blot assay (Figure 2(e)). Compared with the vector group, the expression of cleaved caspase 3 and Bax was increased in PAX8-AS1 group, while the expression of Bcl-2 was decreased. In conclusion, PAX8-AS1 overexpression facilitates the apoptosis of PTC cells, but inhibits cell proliferation.

### 3.3. PAX8-AS1 Binds with miR-96-5p

**Figure 3:**
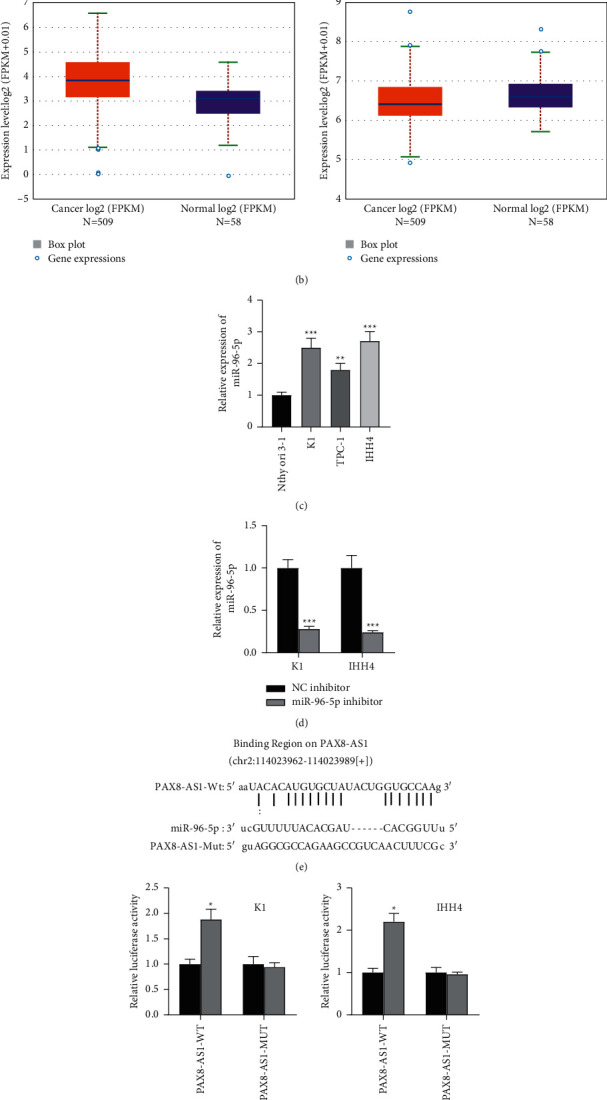
PAX8-AS1 binds with miR-96-5p. *Note.* (a) ENCORI website showed two candidate miRNAs (miR-107 and miR-96-5p) holding binding sites with PAX8-AS1; (b) ENCORI website showed the expression of miR-96-5p and miR-107 in THCA tissues compared to normal tissues; (c) the expression of miR-96-5p in PTC cells compared to normal cells by qRT-PCR; (d) the transfection efficiency of miR-96-5p inhibitor in PTC cells was examined by qRT-PCR; (e) ENCORI website showed a binding region of miR-96-5p on PAX8-AS1; (f) the binding relationship of PAX8-AS1 and miR-96-5p was validated by luciferase reporter assay; ^*∗*^*P* < 0.05,  ^*∗∗*^*P* < 0.01, and ^*∗∗∗*^*P* < 0.001.

ENCORI website predicted two candidate miRNAs, miR-96-5p, and miR-107, holding binding sites with PAX8-AS1 (Figure 3(a)). Furthermore, ENCORI website showed that miR-96-5p was highly expressed in PTC tissues, whereas miR-107 showed decreased expression in PTC tissues (Figure 3(b)). The expression level of miR-96-5p was detected by qRT-PCR in PTC cells. Compared with normal Nthy-ori 3-1 cells, the expression of miR-96-5p was higher in PTC cell lines (Figure 3(c)). qRT-PCR also confirmed decreased miR-96-5p levels in K1 and IHH4 cells after miR-96-5p knockdown (Figure 3(d)). According to the ENCORI website, there was a specific binding region between PAX8-AS1 sequence and the miR-96-5p sequence (Figure 3(e)). Luciferase reporter assay was conducted to determine the binding sites of miR-96-5p on PAX8-AS1. Compared with the NC inhibitor group, the luciferase activity of plasmids containing wildtype PAX8-AS1 (PAX8-AS1-WT) was significantly increased, while the luciferase activity of plasmids containing mutant type PAX8-AS1 (PAX8-AS1-MUT) showed no significant difference after downregulating miR-96-5p (Figure 3(f)). These results indicated that PAX8-AS1 binds with miR-96-5p in PTC cells.

### 3.4. miR-96-5p Downregulation Suppresses PTC Cell Proliferation and Promotes Apoptosis

**Figure 4:**
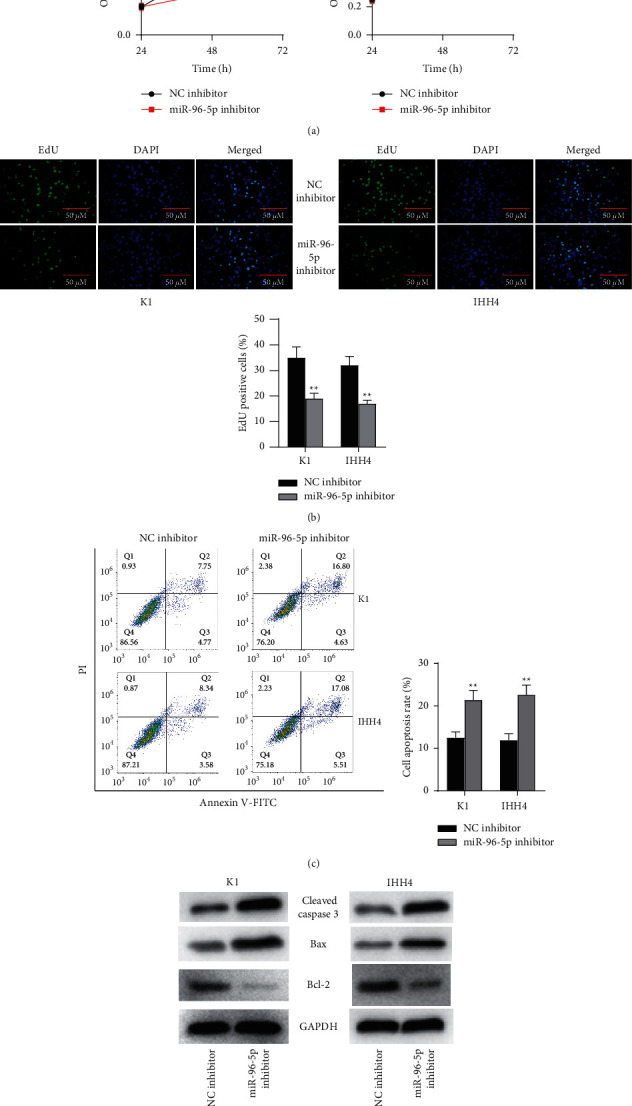
miR-96-5p downregulation suppresses PTC cell proliferation and promotes apoptosis. *Note.* (a) MTT assay was conducted to separately detect the optical density values at 490 nm at 24, 48, and 72 h in K1 and IHH4 cells after downregulating miR-96-5p; (b) cell proliferation was examined in K1 and IHH4 cells after miR-96-5p downregulation by EdU assay; (c) Flow cytometry was used to analyze the scatter patterns of cell cycle distribution of K1 and IHH4 cells after downregulating miR-96-5p; (d) the protein bands of apoptosis-related proteins in K1 and IHH4 cells after downregulating miR-96-5p were detected by western blot. ^*∗∗*^*P* < 0.01.

Since miR-96-5p was discovered to be downregulated in PTC and PAX8-AS1 could bind with miR-96-5p in PTC cells, we knocked down miR-96-5p in PTC cells to investigate the influence of miR-96-5p on PTC cell proliferation and apoptosis. As shown by MTT assay (Figure 4(a)) and EdU assay (Figure 4(b)), the proliferation of K1 and IHH4 cells was suppressed after downregulating miR-96-5p. Furthermore, flow cytometry analysis demonstrated that miR-96-5p knockdown enhanced the apoptosis of K1 and IHH4 cells (Figure 4(c)), followed by increased expression of cleaved caspase 3 and Bax as well as decreased expression of Bcl-2 (Figure 4(d)). Overall, miR-96-5p downregulation represses PTC cell proliferation and promotes apoptosis.

### 3.5. miR-96-5p Regulates the Expression of PKN2

**Figure 5:**
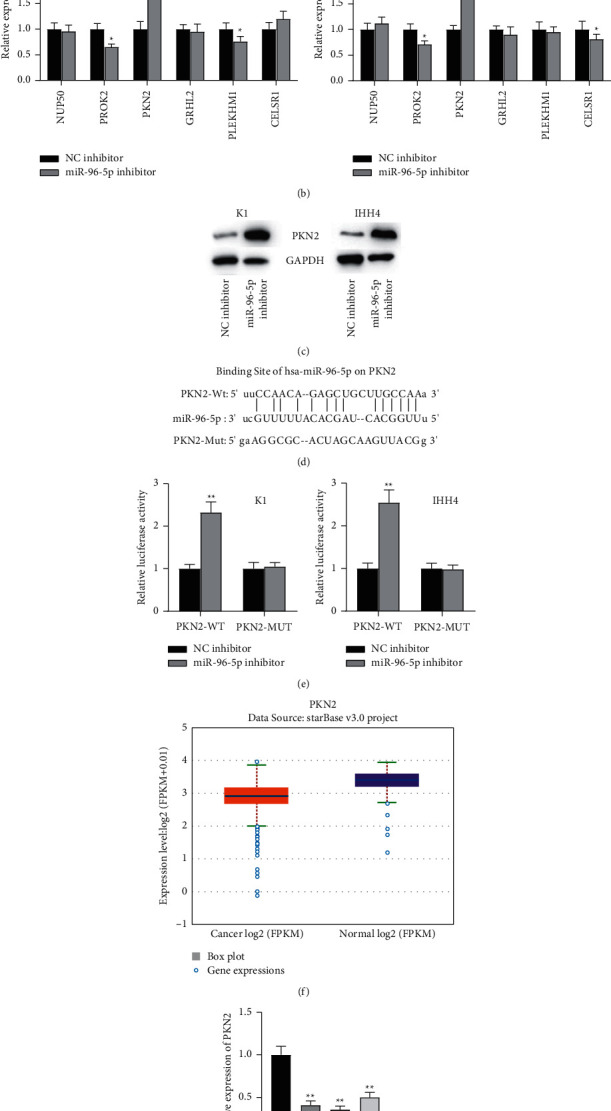
miR-96-5p targets PKN2. *Note.* (a) ENCORI website showing six candidate target mRNAs of miR-96-5p; (b) the expression of six candidate mRNAs in PTC cells after downregulating miR-96-5p; (c) the protein level of PKN2 in PTC cells after miR-96-5p downregulation was detected by western blot; (d) TargetScan showed the binding region of miR-96-5p on PKN2; (e) the binding relationship between PKN2 and miR-96-5p was validated by luciferase reporter assay; (f) ENCORI website showed the expression of PKN2 in THCA tissues compared to normal tissues; (g) the expression of PKN2 in PTC cell lines compared to normal cells was assessed by qRT-PCR. ^*∗*^*P* < 0.05,  ^*∗∗*^*P* < 0.01, and ^*∗∗∗*^*P* < 0.001.

Base on ENCORI website, 6 mRNAs were predicted to hold binding sites with miR-96-5p (Figure 5(a)). Among all these target genes, PKN2 showed the highest relative expression in K1 and IHH4 cells transfected with miR-96-5p inhibitors (Figure 5(b)). Importantly, in K1 and IHH4 cells, the miR-96-5p inhibitor group showed prominently increased mRNA and protein expression of PKN2, compared with the NC inhibitor group (Figure 5(c)). TargetScan (http://www.targetscan.org/) was used to show the binding site of miR-96-5p on PKN2 (Figure 5(d)). Based on the luciferase reporter assay, plasmids containing 3′UTR of wild or mutant fragments from PKN2 (PKN2-WT or PKN2-MUT) and miR-96-5p inhibitor were both cotransfected into the K1 and IHH4 cells. Compared with NC inhibitor group, the luciferase activity of vectors containing PKN2-WT was significantly increased. However, compared with the NC inhibitor group, there was no significant difference in luciferase activity of vectors containing PKN2-MUT after downregulating miR-96-5p (Figure 5(e)). ENCORI website shows that PKN2 has low expression in PTC tissues (Figure 5(f)). qRT-PCR was employed to detect relative expression of PKN2 in PTC cells. Decreased expression level of PKN2 was shown in PTC cells compared with human normal thyroid Nthy-ori3-1 cells (Figure 5(g)). All the above results suggested that miR-96-5P targets PKN2 in PTC.

### 3.6. Interference with PKN2 Reverses the Effect of PAX8-AS1 Overexpression on PTC Cell Proliferation and Apoptosis

**Figure 6:**
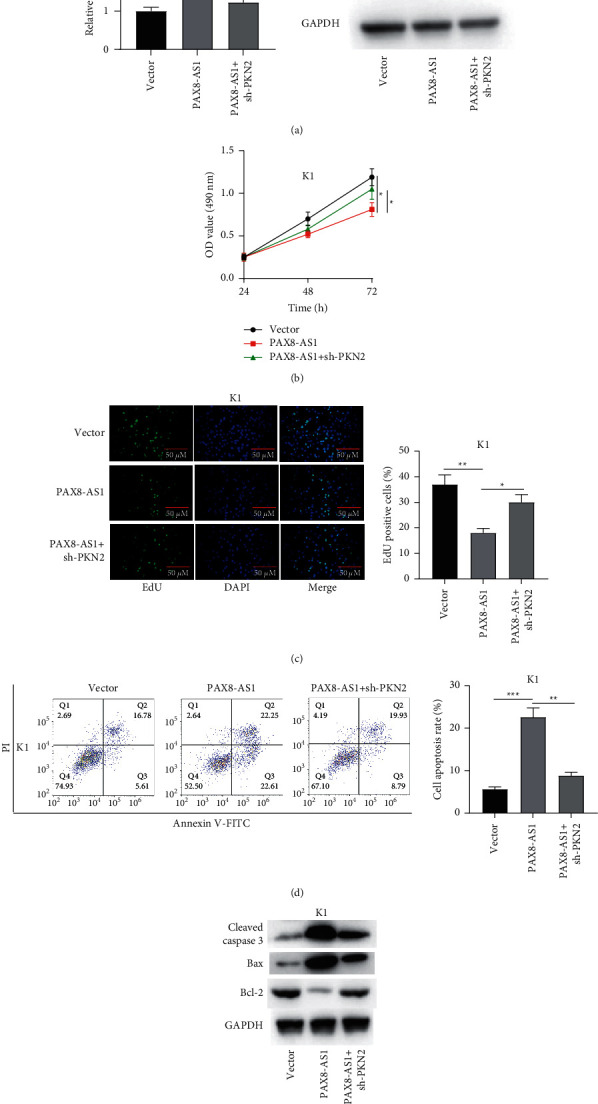
Interference with PKN2 reverses the effect of overexpression of PAX8-AS1 on cell proliferation and apoptosis. *Note.* (a) The expression of PKN2 in PTC K1 cells in vector, PAX8-AS1, and PAX8-AS1+sh-PKN2 groups was detected by qRT-PCR and western blotting; (b) the optical density values at 490 nm at 24, 48, and 72 h in PTC K1 cells in the above three groups were detected by MTT assay; (c) cell proliferation of PTC K1 cells in the above three groups was examined by EdU assay; (d) flow cytometry was used to analyze the scatter patterns of cell cycle distribution of K1 cells in the above three groups; (e) the protein bands of apoptosis-related proteins in K1 cells in the above three groups were detected by western blot. ^*∗*^*P* < 0.05,  ^*∗∗*^*P* < 0.01,  and ^*∗∗∗*^*P* < 0.001.

To confirm how the interaction between PKN2 and PAX8-AS1 regulates PTC development, PKN2 was downregulated in PTC cells transfected with pcDNA3.1/PAX8-AS1. The elevated PKN2 mRNA and protein levels in response to PAX8-AS1 overexpression was reversed in PTC cells cotransfected with PAX8-AS1 and sh-PKN2 (Figure 6(a)). We then tested how PKN2 knockdown influenced the proliferation and apoptosis of PTC K1 cells transfected with pcDNA3.1/PAX8-AS1. Through MTT assay, we determined that the enhanced proliferation of K1 cells in response to PAX8-AS1 overexpression was partially reversed after PKN2 downregulation (Figure 6(b)). The same situation occurred in EdU assay (Figure 6(c)). Through flow cytometry, higher apoptosis rate was discovered in the PAX8-AS1 group compared to the vector group, while the apoptosis rate was reduced in the PAX8-AS1+sh-PKN2 group compared with the PAX8-AS1 group (Figure 6(d)). Compared with the vector group, the PAX8-AS1 group displayed increased protein levels of cleaved caspase 3 and Bax, as well as decreased level of Bcl-2 in K1 cells. However, knockdown of PKN2 partially reversed the effects of PAX8-AS1 overexpression on apoptosis-associated protein levels (Figure 6(e)). These results suggested that interfering with PKN2 reverses the effects of PAX8-AS1 overexpression on PTC cell proliferation and apoptosis.

## 4. Discussion

PTC is one of the most common endocrine malignancies worldwide, and the incidence has been increasing rapidly in the past decade [29]. Although patients with PTC carry a relatively favorable prognosis, some cases still have a risk of developing into more aggressive and fatal cancers because of the metastasis of tumors [30, 31]. High throughput sequencing technology is developing rapidly in recent years, so that many lncRNAs have been discovered, and numerous research studies have demonstrated that lncRNAs are dysregulated in various cancers [32]. The molecules affected are known as tumor suppressors or oncogenes [33]. LncRNAs are RNA molecules whose transcripts are longer than 200 nt and have no ability to code protein [34]. The roles of PAX8-AS1 in different type of cancers as tumor suppressor have been discussed previously [17, 18], which indicated that PAX8-AS1 was downregulated in tumor cells. In this study, the PAX8-AS1 expression was also found to be downregulated in PTC tissues and cell lines, which was in line with existent results, suggesting that PAX8-AS1 may serve as a tumor suppressor in PTC development.

Tumor cells were known as a group of cells with the characteristics of uncontrolled growth and increased migration and invasion ability [35]. Previously, PAX8-AS1 was reported to be downregulated in breast cancer, and PAX8-AS1 can be activated by baicalein to sponging miR-17-5p and upregulating the expression of target genes, therefore inhibiting cell proliferation and promoting apoptosis in breast cancer [36]. This demonstrated that PAX8-AS1 could serve as a potential therapeutic target for breast cancer. In our study, we also found that PAX8-AS1 was downregulated in PTC, and overexpression of PAX8-AS1 inhibited the proliferation and promoted the apoptosis of PTC cells, which were consistent with the previous study. This indicated that PAX8-AS1 could exert the antitumor effect on PTC cell malignant behaviors, suggesting that PAX8-AS1 may serve as a potential therapeutic target for PTC.

In previous studies, lncRNAs serve as a ceRNA or a molecular sponge in the modulation of miRNAs, and then regulate the expression of target mRNAs in the development of many human diseases, including multiple cancers [37]. Such ceRNA network is very common in PTC [38–40]. With the development of the bioinformatics, the target miRNAs of lncRNAs can be predicted by various methods. In the current study, miR-96-5p has been identified as a target miRNA of PAX8-AS1 based on online tools ENCORI. Through literate research, we found that miR-96-5p functions as an oncogene in different type of cancers [23, 41, 42]. Therefore, we hypothesized that PAX8-AS1 may exert its antitumor function by sponging miR-96-5b, thus preventing the inhibition of the miRNA to its target gene. To verify this hypothesis, the relationship between PAX8-AS1 and miR-96-5p was further confirmed. Our study found for the first time that PAX8-AS1 could bind with miR-96-5p, supplementing the underlying mechanism by which PAX8-AS1 mediates the tumorigenesis of PTC. Furthermore, miR-96-5p was previously reported to be upregulated in PTC tissues and cells, and miR-96-5p overexpression promotes the proliferation, invasion, and migration of PTC cells via targeting CCDC67, which has demonstrated that miR-96-5p can act as a potential therapeutic target for PTC [26]. In the current study, we also discovered that miR-96-5p downregulation inhibited PTC cell proliferation and facilitated cell apoptosis, which further validated the role of miR-96-5p as a potential therapeutic target for PTC.

Protein kinase N2 (PKN2) is a PKC-associated serine/threonine-protein kinase and act as a chemical tool to explore several types of cancer [43]. The existent study of the function of PKN2 in tumor is relatively controversial. Particularly, PKN2 was reported as an oncogene in oral squamous cell carcinoma progression [44]. On the contrary, PKN2 was identified to inhibit tumor-associated macrophages (TAMs) polarization and tumor growth in colon cancer [45]. In addition, PKN2 demonstrated a tumor suppressor profile in breast cancer [46]. We also found a decreased mRNA expression of PKN2 in PTC tumor cells, as compared with human normal thyroid cells. Moreover, we validated that miR-96-5p directly targeted and negatively regulated the PKN2 level. As expected, knockdown of PKN2 partially abrogated the antitumor effects of PAX8-AS1 overexpression on PTC cells. Taken together, these results demonstrated that overexpression of PAX8-AS1 may inhibit the development of PTC by sponging miR-96-5p and therefore upregulating PKN2.

To sum up, PAX8-AS1 may function as a tumor suppressor to inhibit the proliferation and enhance the apoptosis of PTC cells via the miR-96-5p/PKN2 axis. Our data suggest that overexpression of PAX8-AS1 inhibits the progression of PTC *in vitro*. Thus, PAX8-AS1 may be a potential target for the treatment of PTC via the miR-96-5p/PKN2 axis.

## Figures and Tables

**Table 1:** The primer sequences.

Gene	Primer sequences
PAX8-AS1	Forward: 5′-TACTGTCAAGGCTGACTGC-3′
	Reverse: 5′-CTCAGAGGAGAAACCAGCC-3′

miR-96-5p	Forward: 5′-ATGCTTTCTCAACTTGTTGG-3′
	Reverse: 5′-TCACCGCTCTTGGCCGTCACA-3′

U6	Forward: 5′-ATACAGAGAAAGTTAGCACGG-3′
	Reverse: 5′-GGAATGCTTCAAAGAGTTGTG-3′

NUP50	Forward: 5′-AAACAACATAACCAGTGCCC-3′
	Reverse: 5′-GCAGCAAGAGAACCAAAGG-3′

PROK2	Forward: 5′-TATGGGCAAACTGGGAGAC-3′
	Reverse: 5′-TTCCTGCCTTCCATTTCCA-3′

PKN2	Forward: 5′-ACTTTCAGAAGCTCAAGCA-3′
	Reverse: 5′-GGACTTCGTTTAATCTTTGCTC-3′

GRHL2	Forward: 5′-ATGATGAACGAGAAGGTGG-3′
	Reverse: 5′-CTCACGTACAAGAGCACTC-3′

PLEKHM1	Forward: 5′-CACCTCATTGGGAGGAGAC-3′
	Reverse: 5′-ATTCCTGTGGTTGAGCCTC-3′

CELSR1	Forward: 5′-CTGTGTCTGATGGCATCCA-3′
	Reverse: 5′-CAGTGATGCTGTTGGTCAG-3′

GAPDH	Forward: 5ʹ-GCATCCTGGGCTACACTG-3ʹ
	Reverse: 5ʹ-TGGTCGTTGAGGGCAAT-3ʹ

## Data Availability

The datasets used or analyzed during the current study are available from the corresponding author on reasonable request.
